# Epigenomic profiling of preterm infants reveals DNA methylation differences at sites associated with neural function

**DOI:** 10.1038/tp.2015.210

**Published:** 2016-01-19

**Authors:** S Sparrow, J R Manning, J Cartier, D Anblagan, M E Bastin, C Piyasena, R Pataky, E J Moore, S I Semple, A G Wilkinson, M Evans, A J Drake, J P Boardman

**Affiliations:** 1 MRC Centre for Reproductive Health, University of Edinburgh, Queen's Medical Research Institute, Edinburgh, UK; 2 MRC Centre for Regenerative Medicine, University of Edinburgh, Edinburgh, UK; 3 University/BHF Centre for Cardiovascular Science, University of Edinburgh, Edinburgh, UK; 4 Centre for Clinical Brain Sciences, University of Edinburgh, Edinburgh, UK; 5 Clinical Research Imaging Centre, University of Edinburgh, Edinburgh, UK; 6 Department of Radiology, NHS Lothian, Edinburgh, UK; 7 Department of Pathology, NHS Lothian, Edinburgh, UK

## Abstract

DNA methylation (DNAm) plays a determining role in neural cell fate and provides a molecular link between early-life stress and neuropsychiatric disease. Preterm birth is a profound environmental stressor that is closely associated with alterations in connectivity of neural systems and long-term neuropsychiatric impairment. The aims of this study were to examine the relationship between preterm birth and DNAm, and to investigate factors that contribute to variance in DNAm. DNA was collected from preterm infants (birth<33 weeks gestation) and healthy controls (birth>37 weeks), and a genome-wide analysis of DNAm was performed; diffusion magnetic resonance imaging (dMRI) data were acquired from the preterm group. The major fasciculi were segmented, and fractional anisotropy, mean diffusivity and tract shape were calculated. Principal components (PC) analysis was used to investigate the contribution of MRI features and clinical variables to variance in DNAm. Differential methylation was found within 25 gene bodies and 58 promoters of protein-coding genes in preterm infants compared with controls; 10 of these have neural functions. Differences detected in the array were validated with pyrosequencing. Ninety-five percent of the variance in DNAm in preterm infants was explained by 23 PCs; corticospinal tract shape associated with 6th PC, and gender and early nutritional exposure associated with the 7th PC. Preterm birth is associated with alterations in the methylome at sites that influence neural development and function. Differential methylation analysis has identified several promising candidate genes for understanding the genetic/epigenetic basis of preterm brain injury.

## Introduction

Preterm birth affects 5–13% of newborns,^1^ and is a profound early-life stressor that is closely associated with cerebral palsy, cognitive impairment, autism spectrum disorder and psychiatric disease.^2, 3, 4, 5, 6^ The prevalence of impairment is related to gestational age at birth and to adverse exposures such as inflammation, ischaemia, respiratory morbidity and sub-optimal nutrition,^7^ but the mechanisms underlying these associations are poorly understood.

Epigenetic modification has a fundamental role in regulating gene expression and determining neural cell fate, and DNA methylation (DNAm) is one such modification that is highly conserved across species.^8^ DNAm is dynamic during development, including in the brain^9^ and this could provide a mechanism by which environmental factors lead to disturbances of neural development that underpin later impairment.^10^ DNAm mediates gene–environment interactions between early-life stress and several neuropsychiatric outcomes,^11, 12, 13, 14^ but little is known about DNAm in relation to brain development after preterm birth.

Although DNAm patterns are tissue specific, a number of recent observations suggest consistency between peripheral tissues and brain. First, DNAm profiles are altered consistently between prefrontal cortex and T cells in a rhesus macaque model of early-life stress.^15^ Second, the top enriched biological processes from peripheral blood cells of adults with post-traumatic stress disorder and early-life trauma concern central nervous system development,^12^ which suggests considerable overlap between tissues. Third, inter-individual variation tends to be consistent across tissue types.^16^ Furthermore, sampling DNA from saliva rather than blood is informative in brain DNAm studies because: methylation profiles obtained from saliva show greater correspondence with brain tissue extracts than those obtained from blood;^17^ inherent properties of DNAm from buccal cells (greater enrichment of DNaseI hypersensivity sites, histone modifications and disease-associated single nucleotide polymorphisms (SNPs)) may make them a more favourable proxy tissue than blood for epigenome-wide association studies of non-haematological disease.^18^

Structural and diffusion magnetic resonance imaging (dMRI) reveal a cerebral signature of preterm birth that includes reduced connectivity of white matter tracts, focal tissue volume reduction in deep grey matter nuclei and reduced cortical complexity.^19, 20, 21, 22, 23, 24^ Specifically, fractional anisotropy (FA) and mean diffusivity (〈D〉) derived from dMRI provide measures of tract integrity in the newborn brain that have a predictable pattern of alteration in preterm infants at term equivalent age (TEA).^25, 26, 27^ These biomarkers are sensitive to genetic and environmental risk modulators for injury, and can detect neuroprotective treatment effects.^28, 29, 30, 31^

Probabilistic neighbourhood tractography (PNT) is an automatic segmentation technique, based on single seed point tractography, that can identify the same fasciculus-of-interest across a group of subjects by modelling how individual tracts compare with a predefined reference tract in terms of length and shape.^32, 33^ This tract shape modelling is unique to PNT and allows not only measurement of tract integrity parameters, such as tract-averaged 〈D〉 and FA, but also provides a metric, the absolute goodness-of-fit of the segmented tract to the reference (*R*), which can be used to quantify differences in tract shape between individuals. The inclusion of anatomic information in dMRI models makes PNT ideally suited to studies of genetic and epigenetic effects because brain structure is heritable.^34, 35^

In this study, we tested the hypotheses that the stress of preterm birth leads to alterations in the methylome that are apparent early in the newborn period, and variance in DNAm is associated with dMRI parameters in major white matter tracts and clinical risk factors for adverse outcome.

## Materials and methods

### Participants

The study was conducted according to the principles of the Declaration of Helsinki, and ethical approval was obtained from the UK National Research Ethics Service. Written parental informed consent was obtained.

The cohort consisted of two groups of neonates who received care at the Royal Infirmary of Edinburgh between January 2012 and September 2014: (1) preterm neonates (defined as postmenstrual age (PMA) at birth <32 completed weeks gestation); and control infants born at full term (>37 weeks PMA). Infants were not eligible if they had dysmorphic features suggestive of a chromosomal abnormality that was confirmed by karyotype, a congenital malformation or a congenital infection.

### DNA extraction

The DNA OG-575 kit was used for sampling of saliva at TEA, defined as 38–42 weeks PMA (DNAGenotek, Ottawa, ON, Canada). DNA was extracted using an alcohol precipitation technique as per manufacturer's instructions, and was rehydrated in TE 0.5. Gel electrophoresis was used to qualify DNA extraction and Qubit 2.0 Fluorometer was utilised for quantification of DNA concentration (Invitrogen Life Sciences, Carlsbad, CA, USA).

### DNAm analysis

DNAm analysis was performed at the Genetics Core of the Edinburgh Clinical Research Facility (Edinburgh, UK). Bisulphite conversion of 500 ng input DNA was carried out using the EZ DNAm Kit (Zymo Research, Freiburg, Germany). Four microlitres of bisulphite-converted DNA was processed using the Infinium HD Assay for Methylation (Illumina Methylation 450k beadchip and Infinium chemistry (Illumina, San Diego, CA, USA)). Each sample was interrogated on the arrays against 485 000 methylation sites. The arrays were imaged on the Illumina HiScan platform and genotypes were called automatically using GenomeStudio Analysis software version 2011.1 (Illumina). The data discussed in this publication have been deposited in NCBI's Gene Expression Omnibus and are accessible through GEO Series accession number GSE72120 (http://www.ncbi.nlm.nih.gov/geo/query/acc.cgi?acc=GSE72120).

### MRI acquisition

The preterm infants underwent brain MRI at TEA. A Siemens MAGNETOM Verio 3T MRI clinical scanner (Siemens Healthcare, Erlangen, Germany) and 12-channel phased-array head coil were used to acquire: T1-weighted MPRAGE volume (~1 mm^3^ resolution), T2-weighted STIR (~0.9 mm^3^ resolution), T2-weighted FLAIR (~1 mm^3^ resolution) and dMRI (11 T2- and 64 diffusion encoding direction (*b*=750 s mm^−^^2^) single-shot spin-echo echo planar imaging volumes with 2 mm isotropic voxels using a prototype sequence). All examinations were supervised by a pediatrician experienced in MRI procedures. Infants were examined in natural sleep and pulse oximetry, temperature and electrocardiography data were monitored throughout. Ear protection was used for each infant, comprising earplugs placed in the external ear and neonatal earmuffs (MiniMuffs, Natus Medical, San Carlos, CA, USA).

### Methylation analysis

Data were processed with the RnBeads tool^36^ v 0.99.17, for processes including data loading, pre-processing, normalisation and differential methylation. The software was modified slightly to allow filtering of problematic CpG loci identified by Chen *et al.*^37^

During pre-processing and using the Chen annotation, probes were removed if their CpG loci overlapped with known SNPs from the 1000 Genomes Project (www.1000genomes.org), if a SNP occurred at the site of single-base extension, or if the probe had been shown to be non-specific. A further, smaller set of probes was removed where RnBeads' iterative ‘GreedyCut' algorithm identified a large number of unreliable measurements across samples.

Normalisation was carried out using the beta mixture quantile dilation method of Teschendorff *et al.*^38^ in which the differing distributions of Type I and Type II probes is taken into consideration. This method had been shown to perform well in comparison with other methods.^39^ Following normalisation a batch correction was applied by use of ComBat^40^ to account for the well-documented chip-wise batch effect of the Infinium platform. Before downstream analyses, probes were removed if their target sites occurred on sex chromosomes, or in non-CpG contexts.

Finally, differential methylation between term and preterm individuals was assessed in gene bodies and promoters. RnBeads includes gene-level annotations from Ensembl (www.ensembl.org; v77 for the version of RnBeads used), and assigns promoters as the regions from 1.5 kb upstream to 0.5 kb downstream of the transcription start site. Differentially methylated positions were assessed with Limma,^41^ and aggregated for genes and promoters using a generalisation of Fisher's method. The false discovery rate (FDR)-corrected version of these aggregated region-level *P*-values was used to select genes with significantly differentially methylated regions (DMR) in bodies and/or promoters. Gene function annotation was determined from the National Center for Biotechnology Information Gene database (http://www.ncbi.nlm.nih.gov/gene/about-generif).

### Validation by pyrosequencing

Pyrosequencing was used to validate DNAm at five selected genes that showed differential methylation (*P*<0.05, FDR corrected) between preterm infants at TEA and term controls: *SLC7A5, SLC1A2, NPBWR1* and *QPRT*. *APOL1* was included in validation studies because of its functional relevance and the significance value from the array was marginal (*P*=0.05). Bisulphite conversion was performed on 500 ng of genomic DNA with the EZ DNAm kit (Zymo Research, Freiburg, Germany). The converted DNA was amplified using the AmpliTaq Gold 360 kit (Applied Biosystems, Warrington, UK) with primers mapping to target regions containing CpGs assayed within the array. PCR primers were designed using PyroMark Assay Design Software 2.0 (Qiagen; https://www.qiagen.com). Pyrosequencing was performed using PyroMark Q24Gold reagents on a PyroMark Q24 Pyrosequencer (Qiagen) according to the manufacturer's instructions. Data were extracted and analysed using PyroMark Q24 1.0.10 software (Qiagen). Background non-conversion levels were ~1–3%.

### Diffusion MRI analysis

After conversion from DICOM to NIfTI-1 format, the dMRI data were preprocessed using FSL tools (http://www.fmrib.ox.ac.uk/fsl) to extract the brain and eliminate bulk patient motion and eddy current-induced artifacts by registering the diffusion-weighted to the first T2-weighted echo planar imaging volume of each subject. Using DTIFIT, 〈D〉 and FA volumes were generated for every subject. From the underlying white matter connectivity data, eight major white matter fasciculi (genu and splenium of corpus callosum, left and right cingulum cingulate gyrus, left and right corticospinal tracts (CST), and left and right inferior longitudinal fasciculi) were identified using PNT optimised for neonatal dMRI. As described in detail in the study by Anblagan *et al.,*^33^ this optimisation principally involved using reference tracts created from a group of 20 term controls.

### Principal components analysis

Dimension reduction using principal components (PC) analysis was used to inspect the dataset for signal in the methylation values that is related to clinical variables and imaging features that are associated with neurodevelopmental outcome (implemented in RnBeads). The clinical variables tested were: gender, PMA at birth, PMA at scan, chorioamnionitis, exposure to antenatal steroids, exposure to antenatal magnesium sulphate, number of days requiring parenteral nutrition and one/more episodes of late-onset sepsis. The image features tested were tract-averaged FA, tract-averaged 〈D〉, and *R* for the eight major fasciculi. Properties of the dataset, which included coordinates in the PC space, clinical variables and image features were tested for association: if both properties contained categorical data, a two-sided Fisher's exact test was used; if both properties contain numerical data the correlation coefficient between the traits was computed, and a *P*-value was estimated using permutation tests with 10,000 permutations; and if property *A* was categorical and property *B* was numeric then the *P*-value for association was calculated by comparing the values of *B* for the different categories in *A (*two-sided Wilcoxon rank sum test when *A* defines two categories, or a Kruskal–Wallis one-way analysis of variance if *A* separates the samples into three or more categories. Because 33 variables were tested for each PC, *P*-values were corrected using FDR, and values <0.05 were considered significant.

### Methylation as a function of clinical/imaging variables

Variables indicated as interesting via PCA were modelled directly using Limma (http://bioinf.wehi.edu.au/limma/), with methylation as a function of the variable in question.

## Results

### Participants

We collected genomic DNA from 36 sex-matched preterm infants (mean PMA at birth 28^+3^ weeks, range 23^+2^–32^+6^; mean birth weight 1057 g, range 568–1460) at TEA (mean PMA 39^+5^ weeks, range 38–42^+4^ weeks), and from 36 sex-matched controls born at term (mean PMA 40^+0,^ range 38^+1^–42^+0^). Seventy out of 72 mothers (97%) reported taking folic acid supplements around the time of conception to at least 12 weeks gestation.

Of the preterm infants, 9 (25%) had intrauterine growth restriction, 35 (97%) had been exposed to antenatal steroids, 20 (56%) had been exposed to antenatal MgSO_4_ and 11 (31%) had histological chorioamnionitis. The mean duration requiring parenteral nutrition after birth was 11 days (range 5–25).

Of the controls, none had intrauterine growth restriction, and none was exposed to MgSO_4_ or steroids for threatened preterm labour at any stage in pregnancy. None received parenteral nutrition.

The mean (range) DNA yield was 45.9 ng μl^−1^ (13.4–95.9) from preterm infants and 36.35 ng μl^−1^ (8.12–80) from term infants.

### Association between DNAm and preterm birth

About 112,818 probes were removed after: first, pre-filtering (probes on SNPs (*n*=66,877); non-specific probes (*n*=26,505); sites with excess high detection *P*-values (*n*=8,852)); and second, post-filtering (non-CpG probes (*n*=1130); and probes on sex chromosomes (*n*=9,454)). The remaining probes were used to calculate aggregate *P*-values for DMRs in two categories: gene bodies and promoters. About 87 genes were assigned as differentially methylated by this approach (*P*<0.05, FDR corrected, Supplementary Table 1), of which 25 were protein coding. About 138 promoter regions were differentially methylated (Supplementary Table 2), of which 58 related to protein-coding genes. About 34 genes were present in both sets (partly due to the overlapping gene and promoter definitions), of which 11 were protein coding. Genes that encode proteins with neural function and/or those with neuropsychiatric disease associations are listed in Table 1.

### Array validation

**Figure 1:**
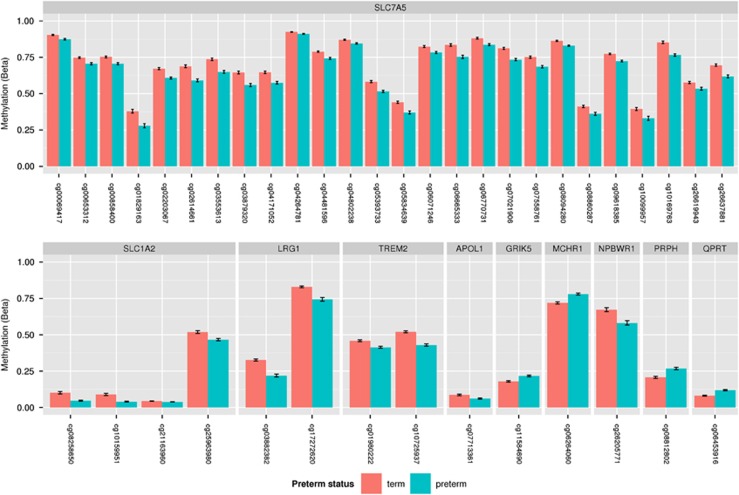
Differential methylation between preterm infants at term equivalent age and healthy infants born at term at CpG sites in protein-coding genes with neural function identified in the array (*P*<0.05, corrected).

To validate the array findings, pyrosequencing was performed at selected annotated CpG sites in five selected genes: *SLC7A5, SLC1A2, NPBWR1, APOL1* and *QPRT.* CpG sites in all five genes which were identified on the array were confirmed as being differentially methylated (Figure 1, Table 2). Because some assays covered additional neighbouring CpGs, which were not also interrogated in the array, it was possible to assess methylation patterns in the nearby region. For *SLC7A5*, the assay covered three upstream CpGs that all showed similar differences in methylation; the *SLC7A2* and *APOL1* assays both captured a second neighbouring downstream CpG that was also differentially methylated; and the assay for *NPBWR1* cg26205771 covered 1 upstream and 1 downstream CpG, and both showed similar methylation patterns.

### Diffusion MRI analysis

**Figure 2:**
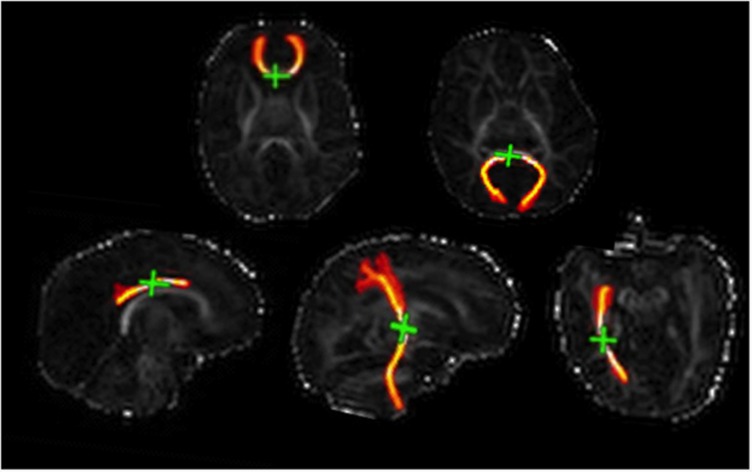
Illustration of segmented tracts overlaid on FA maps. Top row: genu (left) and splenium (right) of corpus callosum. Bottom row (right to left): left CCG, right CST and right ILF. CCG, cingulum cingulate gyrus; CST, corticospinal tract; FA, fractional anisotropy; ILF, inferior longitudinal fasciculus.

Figure 2 shows illustrations of segmented tracts for a representative subject, while Table 3 presents descriptive statistics for 〈D〉, FA and *R* in the eight major fasciculi identified from the dMRI data in the preterm group using PNT.

### Principal components analysis

Ninety-five percent of the variance in the preterm methylome was explained by 23 components, with most variance explained by the first two PCs (31.8% and 20.1%, respectively), (Supplementary Table 3). In exploratory analyses, gender was associated with the first PC (*P*=0.0071); FA in the genu of the corpus callosum was associated with the 5th PC (3.3% variance; *P*=0.0061); right CST *R* and chorioamnionitis were associated with the 6th PC (2.9% variance; *P*=0.0011 and *P*=0.0053, respectively); and both gender (*P*=0.0016) duration of parenteral nutrition use (*P*=0.0017) were associated with the 7th PC (2.6% variance). After correction for multiple tests, three associations remained: right CST *R* with the 6th PC (*P*=0.036); and both gender (*P*=0.028) and duration of parenteral nutrition (*P*=0.028) with the 7th PC. No variable was significantly associated with any DMR when tested directly (adjusted *P*-value<0.05).

## Discussion

In a deeply phenotyped representative sample of newborns, preterm birth was associated with significant alterations in the methylome in 10 protein-coding genes whose products influence neural cell function and are associated with behavioural traits/neuropsychiatric disease. We found that specific risk modulators of neurodevelopmental outcome after preterm birth (gender, chorioamnionitis and early nutritional factors) explained a modest, but significant proportion of the variance in DNAm. Furthermore, there was an association between DNAm and white matter tract tissue integrity and shape inferred from dMRI, suggesting that epigenetic variation may contribute to the cerebral phenotype of preterm birth.

Epigenome-wide association studies have provided new insights into genes whose regulation pattern varies in the context of child abuse, post-traumatic stress disorder, schizophrenia and autism spectrum disorder,^12, 64, 65^ but to our knowledge this is the first epigenome-wide association studies in preterm infants and healthy controls to identify differential methylation at loci that influence neural development. The magnitude of the DNAm differences between preterm and term infants varied between and within genes; however, in support of a potential biological role for these changes, differential methylation was identified at multiple CpGs on the array for most loci. Pyrosequencing analysis for all genes selected for validation confirmed the differences at individual CpGs seen on the array and also identified additional differentially methylated neighbouring CpGs, suggesting that preterm birth associates with widespread effects on DNAm at these loci. For the majority of these genes, differential DNAm was identified in the gene promoter, although for *LRG1* and *SLC7A5* differential methylation also extended into the gene body. In general, DNAm at DMRs has a negative correlation with gene expression, with recent studies reporting that this correlation is stronger not only for CpGs close to the transcription start site but also for intragenic DMRs, which do not necessarily mark intragenic CpG islands or CGI shores and may instead represent functional elements.^66^

A particularly large number of differentially methylated CpGs were identified in two members of the solute transporter family of membrane transport proteins, *SLC7A5* and *SLC1A2.* In preterm infants, a significant reduction (~10%) in DNAm was seen at multiple CpGs in the *SLC7A5* promoter and gene body on the array, and more were identified on pyrosequencing. *SLC7A5* (also known as *LAT1*) is a member of the solute transporter family of membrane transport proteins and is involved in the transport of large amino acids, including methionine, across the blood–brain barrier.^67^ Methionine is a key component of S-adenosylmethionine, the major methyl donor and very recent data suggests that *SLC7A5* can act as an indirect regulator of the epigenome, at least in terms of histone modification through effects on the availability of methionine and the subsequent availability of S-adenosylmethionine.^68^ *SLC1A2* (also known as *EAAT2, GLT-1*) is predominantly expressed in astrocytes but is also expressed by oligodendroglia and macrophages, and on neurons during development. It has a role for clearing glutamate throughout the neuroaxis and can be both transcriptionally and post-transcriptionally regulated.^44^ The expression of *SLC1A2* is reported to be developmentally regulated, particularly at the window of peak vulnerability for the development of periventricular leukomalacia.^69, 70^

The prevailing form of preterm brain injury is diffuse and involves multiple cell lines (reviewed by Back and Miller^71^). The pathogenesis includes death of pre-myelinating oligodendrocytes (pre-OLs) because of vulnerability to inflammatory mediators, reactive oxygen and nitrogen species, and glutamate excitotoxicity. This is followed by defective pre-OL regeneration and repair, leading to hypomyelination. In pre-clinical and human post-mortem studies the diffuse form of white matter injury coincides with enrichment of reactive glia (activated microglia/macrophages and reactive astrocytes) that inhibit the maturation of pre-OLs to myelin-forming oligodendrocytes. The neuronal population is not thought to degenerate under conditions that generate pre-OL loss (outside the context of tissue necrosis and cystic periventricular leucomalacia), but rather there is a dysmaturation response characterized by aberrant dendritic arborisation, disturbances in synaptic activity and reduced spine density. The functional profiles of the 10 genes that we found to be differentially methylated in preterm infants include neuronal and glial signalling, neurotransmission, apoptosis and cellular energetics. Our findings focus attention on the role of these genes in mediating injury and regeneration/repair processes after preterm birth, and their candidacy is further strengthened by the neuropsychiatric disease associations in later life (Table 1).

PCs analysis was used to explore whether dMRI measures in the major white matter fasciclui or clinical risk factors contributed to structure in the methylation data of the preterm group. After correction for multiple tests and exclusion of probes on sex chromosomes, a small proportion of the variance was explained by the shape of CST, which was associated with the 6th principal component (3.3% of variance); and of the clinical factors tested, gender and number of days requiring parenteral nutrition, both associated with the 7th principal component (2.9% of variance). However, none of these three variables was significantly associated with any DMR when tested directly, which indicates that if an effect is present, it is subtle and distributed over many loci.

We sampled the methylome at a single time point chosen to reflect the allostatic load of preterm birth and neonatal intensive care among children who survive to hospital discharge, but this leaves uncertainty about the temporal cues for epigenetic modification in the perinatal period. A recent study of DNAm in umbilical cord blood of 11 preterm infants and 11 term controls demonstrated 20 DMRs between the groups, including loci in 3 genes that are involved with neuronal development: *PPT2, GABBR1, PLEKHB1.*^72^ We did not identify DMRs in these genes, which may be explained by differences in study population, timing of sampling or tissue-type sampled. A multiple sampling design that includes parental samples, placental tissue, cord blood and extends across the life-course would be required to investigate the relative contributions of *in utero* and postnatal exposures to changes in DNAm, and the extent to which preterm birth leaves a legacy on the methylome.^73^

In conclusion, these novel data show that the profound early-life stress of preterm birth is associated with differential methylation at sites in several protein-coding genes. The analysis of differential methylation has identified provide promising candidate genes for understanding genetic influences on brain development after preterm birth.

## Figures and Tables

**Table 1:** Differential methylation between preterm infants at term equivalent age and healthy controls in protein-coding genes with neural functions and disease associations

*Gene symbol*	*Gene name*	*Function/disease association*	*Position*	*Number of differentially methylated CpGs (*P*<0.05 FDR corrected)*	*Direction of change in preterm infants relative to term infants*
*SLC7A5*	Solute carrier family 7 (amino acid transporter light chain, L system), member 5	L-type amino acid transporter. Determining role in the permeation of branch chain amino acids and amino acid related drugs (L-Dopa) across the blood brain barrier,^42^ and thyroid hormone uptake in foetal cortex^43^	Promoter and gene	26	↓
*SLC1A2*	Solute carrier family 1 (glial high affinity glutamate transporter), member 2	Principal membrane-bound transporter that clears the excitatory neurotransmitter glutamate from the extracellular space at synapses in the central nervous system. Associated with schizophrenia, bipolar disorder, and neurodegeneration^44, 45^	Promoter	4	↓
*NPBWR1*	Neuropeptides B/W receptor 1	Neuropeptide and opioid receptor. Associated with memory function,^46^ eating behaviours^47^ and processing social information^48^	Promoter	1	↓
*APOL1*	Apolipoprotein L, 1	Secreted high density lipoprotein which binds to apolipoprotein A-I. Associated with schizophrenia susceptibility^49, 50^	Gene	1	↓
*QPRT*	Quinolinate phosphoribosyltransferase	Catabolises quinolate, a potent neuronal excitotoxin, and may inhibit apoptosis.^51^ Associated with Alzheimer's disease^52^ and epilepsy^53^	Promoter	1	↑
*LRG1*	Leucine-rich alpha-2-glycoprotein 1	Involved in protein–protein interaction, signal transduction, cell adhesion and development, is expressed in astrocytes of cerebral cortex and is linked with ageing and neurodegeneration^54, 55^	Promoter and gene	2	↓
*PRPH*	Peripherin	Neuronal cytoskeletal protein. Associated with susceptibility to amyotrophic lateral sclerosis^56^ and frontotemporal lobar degeneration^57^	Promoter	1	↑
*GRIK5*	Glutamate receptor, ionotropic, kainate 5	Member of the glutamate-gated ionic channel family. Associated with schizophrenia^58, 59^	Promoter	1	↑
*TREM2*	Triggering receptor expressed on myeloid cells 2	Membrane protein that forms a receptor signalling complex with tyrosine kinase-binding protein. Mutations associated with pre-senile dementia and demyelination^60, 61^	Promoter	2	↓
*MCHR1*	Melanin-concentrating hormone receptor 1	Member of the G protein-coupled receptor family 1, an integral plasma membrane protein that binds melanin-concentrating hormone. The encoded protein can inhibit cAMP accumulation and stimulate intracellular calcium flux. Differential methylation is associated with obesity^62^ and receptor antagonists control obesity and influence mood^63^	Promoter	1	↑

Abbreviation: FDR, false discovery rate.

**Table 2:** Pyrosequencing results for 5 genes (13 CpG sites) that showed differential methylation between groups in the array

*Gene symbol*	*CpG site*	*Term mean % methylation (s.d.)*	*Preterm mean % methylation (s.d.)*	*% Difference (term–preterm)*	P*-value*
*QPRT*	cg06453916	11.4 (1.1)	12.5 (1.5)	−1.1	1.24E−03
*SLC7A5*	−45	77.2 (7.4)	68.9 (8.8)	8.3	8.30E−04
	−39	60.1 (8.2)	50.7 (8.9)	9.4	3.81E−04
	−23	80.0 (5.9)	71.0 (10.9)	9.1	7.68E−04
	cg05834639	36.6 (7.2)	28.6 (8.6)	8.1	8.74E−04
*SLC1A2*	cg25963980	31.2 (4.9)	25.7 (4.65)	5.5	5.94E−06
	+10	41.2 (5.0)	34.4 (6.0)	6.8	2.43E−06
*APOL1*	cg36649144	29.5 (6.8)	24.3 (11.9)	5.2	0.0396
	+9	14.6 (3.6)	11.5 (5.8)	3.14	0.0159
*NPBWR1*	cg07629017	6.0 (2.8)	4.74 (1.3)	1.26	0.0195
*NPBWR1*	−5	64.5 (7.5)	56.5 (10.3)	8.0	4.11E−04
	cg26205771	55.6 (7.1)	48.1 (8.1)	7.5	1.00E−04
	+4	58.4 (8.6)	50.0 (10.4)	8.4	4.57E−04

Positions of additional cytosines covered by pyrosequencing assays but which are not present on the array are given relative to the annotated CpG from the array.

**Table 3:** Mean (s.d.) of tract-averaged FA and 〈D〉, and median (IQR/2) values of *R* for each major fasciculus

	*Genu*	*Splenium*	*Right CST*	*Left CST*	*Right CCG*	*Left CCG*	*Right ILF*	*Left ILF*
FA	0.20 (0.04)	0.26 (0.04)	0.27 (0.03)	0.28 (0.04)	0.20 (0.03)	0.19 (0.03)	0.22 (0.03)	0.19 (0.03)
〈D〉 (*s.d.*)/x10^-3^ mm^2^ s^−1^	1.497 (0.075)	1.586 (0.162)	1.192 (0.077)	1.240 (0.065)	1.375 (0.186)	1.365 (0.067)	1.664 (0.207)	1.685 (0.210)
*R* (IQR/2)	−4.81 (2.27)	−7.73 (4.36)	−2.66 (1.41)	−3.30 (1.71)	−3.21 (2.44)	−2.78 (3.37)	−3.07 (2.67)	−0.97 (1.78)

Abbreviations: CCG, cingulum cingulate gyrus; CST, corticospinal tract; FA, fractional anisotropy; ILF, inferior longitudinal fasciculus; IQR, interquartile range.
