# Supplementary material for: Epigenomic profiling of preterm infants reveals DNA methylation differences at sites associated with neural function
Source: Transl Psychiatry. 2016 Jan 19;6(1):e716–. doi: 10.1038/tp.2015.210 (PMC5068883; doi:10.1038/tp.2015.210)
Supplement: Supplementary Table 1 [file tp2015210x1.pdf]

Table 1: Differentially expressed genes, as derived by RnBeads

Ensembl ID	Chr	Start	End	Symbol	Entrez ID	Description	Gene type	No. sites	No. sites signif	Mean (0)	Mean (1)	Diff	p.fdr	Rank	DM Promoter
ENSG00000224764	chr9	97690833	97697228	RP11-54O15.3			antisense	1	1	0.28	0.19	0.088	0.00098	12	No
ENSG00000260466	chr16	87870138	87871269	RP4-536B24.2			antisense	2	1	0.38	0.28	0.1	0.0019	21	No
ENSG00000136514	chr3	187086120	187089864	RTP4	64108	receptor (chemosensory) transporter protein 4 [Source:HGNC Symbol; Acc:HGNC:23992]	protein coding	4	2	0.68	0.57	0.1	0.00062	31	Yes
ENSG00000250280	chr12	65675437	65680508	RP11-305O6.3			sense intronic	2	1	0.29	0.39	-0.095	0.0045	33	Yes
ENSG00000258935	chr14	91033839	91036320	RP11-1078H9.2			lincRNA	1	1	0.35	0.44	-0.084	0.0019	34	No
ENSG00000136881	chr9	104122699	104145801	BAAT	570	bile acid CoA:amino acid N-acyltransferase [Source:HGNC Symbol; Acc:HGNC:932]	protein coding	1	1	0.53	0.43	0.1	0.00098	35	Yes
ENSG00000129048	chr3	132316081	132337811	ACKR4	51554	atypical chemokine receptor 4 [Source:HGNC Symbol; Acc:HGNC:1611]	protein coding	2	1	0.45	0.53	-0.079	0.0027	39	No
ENSG00000249429	chr5	142869420	142910915	CTD-2050E21.1			lincRNA	1	1	0.39	0.51	-0.11	0.0073	39	No
ENSG00000252774	chr15	64634738	64634897	SNORA48		Small nucleolar RNA SNORA48 [Source:RFAM; Acc:RF00554]	snoRNA	1	1	0.13	0.23	-0.11	0.011	45	Yes
ENSG00000261117	chr2	12855541	12856353	RP11-333O1.1			lincRNA	1	1	0.37	0.3	0.065	0.0018	49	Yes
ENSG00000132530	chr17	6658766	6678966	XAF1	54739	XIAP associated factor 1 [Source:HGNC Symbol; Acc:HGNC:30932]	protein coding	11	6	0.55	0.5	0.056	0.019	62	No
ENSG00000259450	chr15	39480639	39486510	RP11-265N7.1			lincRNA	1	1	0.43	0.51	-0.085	0.0068	64	No
ENSG00000239482	chr3	112021325	112051638	RP11-90K6.1			lincRNA	2	1	0.35	0.42	-0.067	2.9e-05	75	No
ENSG00000164241	chr5	126378250	126409184	C5orf63	401207 101060408	chromosome 5 open reading frame 63 [Source:HGNC Symbol; Acc:HGNC:40051]	protein coding	10	4	0.42	0.47	-0.048	0.01	81	Yes
ENSG00000261550	chr16	53069602	53086785	RP11-467J12.4			antisense	1	1	0.21	0.29	-0.075	0.044	84	No
ENSG00000229167	chr1	32037186	32041174	RP11-73M7.1			antisense	2	1	0.57	0.48	0.087	2.9e-05	86	No
ENSG00000250509	chr5	179868559	179870238	CTC-573N18.1			lincRNA	1	1	0.34	0.26	0.076	0.046	86	Yes
ENSG00000184795	chr11	67479173	67483476	UNC93B5		unc-93 homolog B5 pseudogene (C. elegans) [Source:HGNC Symbol; Acc:HGNC:34051]	unprocessed pseudogene	3	1	0.24	0.2	0.048	0.034	86	No
ENSG00000171236	chr19	4536421	4540486	LRG1	116844	leucine-rich alpha-2-glycoprotein 1 [Source:HGNC Symbol; Acc:HGNC:29480]	protein coding	5	2	0.69	0.62	0.063	0.018	90	Yes
ENSG00000259727	chr15	63188011	63191742	RP11-1069G10.2			lincRNA	1	1	0.54	0.64	-0.095	0.026	95	No
ENSG00000204193	chr9	113065801	113100127	TXNDC8	255220	thioredoxin domain containing 8 (spermatozoa) [Source:HGNC Symbol; Acc:HGNC:31454]	protein coding	2	1	0.43	0.5	-0.072	0.044	107	Yes
ENSG00000258355	chr12	106639391	106640734	RP11-651L5.2			sense intronic	1	1	0.39	0.33	0.053	0.03	125	No
ENSG00000109272	chr4	74718906	74719872	PF4V1	5197	platelet factor 4 variant 1 [Source:HGNC Symbol; Acc:HGNC:8862]	protein coding	6	5	0.32	0.36	-0.047	0.013	126	Yes
ENSG00000255038	chr11	65834748	65837090	RP11-1167A19.2			antisense	2	1	0.42	0.48	-0.062	0.0084	143	No
ENSG00000228176	chr1	30181698	30182394	RP4-656G21.1			lincRNA	1	1	0.43	0.37	0.055	0.04	147	Yes
ENSG00000235285	chr13	44720606	44732358	SMIM2-IT1	100874377	SMIM2 intronic transcript 1 [Source:HGNC Symbol; Acc:HGNC:41492]	sense intronic	1	1	0.55	0.63	-0.079	0.0073	159	No
ENSG00000258987	chr14	94405901	94410475	RP11-131H24.4			antisense	5	2	0.39	0.34	0.041	0.022	162	No
ENSG00000254632	chr11	76470960	76479267	RP11-21L23.4			antisense	1	1	0.65	0.57	0.077	0.012	179	No
ENSG00000261269	chr5	71735730	71738214	RP11-389C8.2			sense overlapping	1	1	0.61	0.69	-0.081	0.0059	182	Yes
ENSG00000230454	chr3	50297735	50300790	U73166.2			lincRNA	3	2	0.34	0.38	-0.037	0.00069	190	No
ENSG00000068079	chr17	41158742	41166473	IFI35	3430	interferon-induced protein 35 [Source:HGNC Symbol; Acc:HGNC:5399]	protein coding	3	2	0.24	0.2	0.037	0.00062	192	Yes
ENSG00000172232	chr19	825097	832017	AZU1	566	azurocidin 1 [Source:HGNC Symbol; Acc:HGNC:913]	protein coding	10	4	0.51	0.47	0.036	0.021	206	No

ENSG00000108448	chr17	18601311	18639431	TRIM16L	147166	tripartite motif containing 16-like [Source:HGNC Symbol; Acc:HGNC:32670]	protein coding	4	1	0.59	0.66	-0.074	0.0026	216	No
ENSG00000066697	chr9	103189438	103213511	MSANTD3	91283	Myb/SANT-like DNA-binding domain containing 3 [Source:HGNC Symbol; Acc:HGNC:23370]	protein coding	3	1	0.23	0.2	0.037	0.026	241	No
ENSG00000188394	chr9	125796806	125797975	GPR21	2844	G protein-coupled receptor 21 [Source:HGNC Symbol; Acc:HGNC:4476]	protein coding	3	1	0.63	0.66	-0.035	0.0029	274	Yes
ENSG00000232799	chr2	210009965	210012411	CRYGFP		crystallin, gamma F pseudogene [Source:HGNC Symbol; Acc:HGNC:2413]	unprocessed pseudogene	5	1	0.75	0.68	0.073	0.05	294	No
ENSG00000260604	chr6	3905144	3912213	RP1-140K8.5			lincRNA	2	1	0.63	0.57	0.057	0.021	298	Yes
ENSG00000162723	chr1	159921282	159924044	SLAMF9	89886	SLAM family member 9 [Source:HGNC Symbol; Acc:HGNC:18430]	protein coding	4	2	0.54	0.59	-0.053	0.0091	311	No
ENSG00000256357	chr14	94896970	94931067	RP11-349I1.2			antisense	4	2	0.66	0.71	-0.053	0.02	313	Yes
ENSG00000269275	chr19	55888947	55889493	CTD-2105E13.15			antisense	4	2	0.7	0.64	0.068	0.0041	315	Yes
ENSG00000182379	chr12	57610578	57620232	NXPH4	11247	neurexophilin 4 [Source:HGNC Symbol; Acc:HGNC:8078]	protein coding	19	9	0.36	0.33	0.03	0.018	326	No
ENSG00000225208	chr10	103070775	103071262	RP11-107I14.2			lincRNA	1	1	0.53	0.59	-0.054	0.0028	334	Yes
ENSG00000232170	chr10	8301294	8310268	LINC00708	100507143	long intergenic non-protein coding RNA 708 [Source:HGNC Symbol; Acc:HGNC:44694]	lincRNA	3	2	0.56	0.61	-0.056	0.044	346	No
ENSG00000103257	chr16	87863629	87903094	SLC7A5	8140	solute carrier family 7 (amino acid transporter light chain, L system), member 5 [Source:HGNC Symbol; Acc:HGNC:11063]	protein coding	50	25	0.61	0.56	0.05	0.0019	359	Yes
ENSG00000248884	chr5	67726254	67730308	CTC-537E7.3			lincRNA	2	1	0.062	0.033	0.028	0.00043	378	Yes
ENSG00000251875	chr1	179545403	179545484	RNU5F-2P		RNA, U5F small nuclear 2, pseudogene [Source:HGNC Symbol; Acc:HGNC:42510]	snRNA	2	1	0.48	0.44	0.043	0.038	386	No
ENSG00000147883	chr9	22002902	22009362	CDKN2B	1030	cyclin-dependent kinase inhibitor 2B (p15, inhibits CDK4) [Source:HGNC Symbol; Acc:HGNC:1788]	protein coding	4	2	0.16	0.13	0.028	0.023	389	No
ENSG00000229257	chr9	139952115	139956913	RP11-229P13.22			antisense	1	1	0.77	0.71	0.064	0.019	442	Yes
ENSG00000258376	chr14	73710726	73712687	RP4-647C14.2			antisense	4	3	0.63	0.58	0.05	0.00098	450	Yes
ENSG00000163874	chr1	37940153	37949980	ZC3H12A	80149	zinc finger CCCH-type containing 12A [Source:HGNC Symbol; Acc:HGNC:26259]	protein coding	12	5	0.36	0.33	0.032	0.026	457	No
ENSG00000206612	chr12	49050431	49050565	SNORA2A	677793	small nucleolar RNA, H/ACA box 2A [Source:HGNC Symbol; Acc:HGNC:32584]	snoRNA	1	1	0.75	0.82	-0.065	0.00098	475	Yes
ENSG00000238279	chr1	153506079	153507591	BX470102.3			antisense	1	1	0.61	0.66	-0.051	0.0051	517	No
ENSG00000160472	chr19	55888204	55889612	TMEM190	147744	transmembrane protein 190 [Source:HGNC Symbol; Acc:HGNC:29632]	protein coding	5	2	0.74	0.69	0.054	0.023	554	Yes
ENSG00000258847	chr14	66424015	66471241	CTD-2014B16.3			lincRNA	3	1	0.68	0.73	-0.054	0.00069	565	No
ENSG00000200354	chr20	37062508	37062641	SNORA71D	677840	small nucleolar RNA, H/ACA box 71D [Source:HGNC Symbol; Acc:HGNC:32657]	snoRNA	1	1	0.78	0.72	0.058	0.0074	573	No
ENSG00000266998	chr17	75369900	75373318	RP11-936I5.1			antisense	9	4	0.59	0.62	-0.023	0.022	603	No
ENSG00000255093	chr11	111322064	111328876	RP11-794P6.2			antisense	1	1	0.62	0.58	0.041	0.0019	726	No
ENSG00000267092	chr19	1508381	1508962	CTB-25B13.9			antisense	2	1	0.13	0.11	0.021	0.03	741	No
ENSG00000249184	chr4	152720652	152722500	RP11-424M21.1			lincRNA	1	1	0.79	0.84	-0.054	0.026	764	Yes
ENSG00000232991	chr2	108370568	108438585	GACAT1		gastric cancer associated transcript 1 (non-protein coding) [Source:HGNC Symbol; Acc:HGNC:48336]	lincRNA	1	1	0.72	0.77	-0.048	0.0021	805	No
ENSG00000108759	chr17	39616063	39623681	KRT32	3882	keratin 32, type I [Source:HGNC Symbol; Acc:HGNC:6449]	protein coding	3	1	0.77	0.82	-0.048	0.0016	903	Yes
ENSG00000259177	chr15	90936722	90941113	RP11-154B12.3			antisense	1	1	0.75	0.8	-0.046	0.0027	931	No
ENSG00000259287	chr15	34150918	34157026	RP11-3D4.2			antisense	1	1	0.74	0.78	-0.044	0.023	972	No

ENSG00000234771	chr9	130873450	130881013	SLC25A25-AS1	100289019	SLC25A25 antisense RNA 1 [Source:HGNC Symbol; Acc:HGNC:27844]	antisense	2	1	0.41	0.44	-0.023	0.04	1172	No
ENSG00000151631	chr10	4914093	4934167	AKR1C6P	389932	aldo-keto reductase family 1, member C6, pseudogene [Source:HGNC Symbol; Acc:HGNC:44680]	unprocessed pseudogene	1	1	0.92	0.87	0.047	0.00065	1206	No
ENSG00000253792	chr5	157600609	157603430	CTC-436K13.5			lincRNA	1	1	0.91	0.87	0.046	0.00029	1232	Yes
ENSG00000253673	chr5	157602404	157627024	CTC-436K13.1			lincRNA	1	1	0.91	0.87	0.046	0.00029	1232	Yes
ENSG00000235700	chr1	157098154	157098463	CYCSP52	360155	cytochrome c, somatic pseudogene 52 [Source:HGNC Symbol; Acc:HGNC:24393]	processed pseudogene	1	1	0.82	0.86	-0.043	0.0011	1257	Yes
ENSG00000260362	chr16	9760742	9770700	RP11-297M9.1			lincRNA	1	1	0.85	0.81	0.042	0.048	1310	No
ENSG00000267239	chr18	13203773	13216366	RP11-794M8.1			antisense	1	1	0.84	0.8	0.041	0.016	1342	No
ENSG00000207625	chr3	35785968	35786051	MIR128-2	406916	microRNA 128-2 [Source:HGNC Symbol; Acc:HGNC:31511]	miRNA	1	1	0.87	0.83	0.039	0.05	1527	No
ENSG00000262097	chr16	14103137	14109873	CTD-2135D7.5			lincRNA	1	1	0.9	0.86	0.038	0.012	1652	No
ENSG00000179447	chr20	19222946	19265240	RP5-1027G4.3	100130264		antisense	1	1	0.83	0.86	-0.037	0.006	1680	No
ENSG00000236171	chr10	118955679	118956165	RP11-501J20.3			processed pseudogene	2	2	0.87	0.83	0.036	0.026	1763	No
ENSG00000231394	chr7	55661470	55662699	RP11-310H4.3			lincRNA	1	1	0.77	0.74	0.031	0.011	1945	Yes
ENSG00000234465	chr19	44080952	44088116	PINLYP	390940	phospholipase A2 inhibitor and LY6/PLAUR domain containing [Source:HGNC Symbol; Acc:HGNC:44206]	protein coding	1	1	0.75	0.78	-0.029	0.041	2127	No
ENSG00000253509	chr8	41132634	41134522	CTD-3080F16.3			sense intronic	1	1	0.76	0.79	-0.03	0.0018	2160	No
ENSG00000249942	chr4	75418301	75514664	AC142293.3			antisense	3	1	0.046	0.057	-0.011	0.041	2197	Yes
ENSG00000208036	chr7	99691616	99691697	MIR106B	406900	microRNA 106b [Source:HGNC Symbol; Acc:HGNC:31495]	miRNA	2	1	0.78	0.81	-0.028	0.029	2462	No
ENSG00000228680	chr7	43278665	43288867	AC004692.4			antisense	6	4	0.71	0.69	0.018	0.021	2651	Yes
ENSG00000178395	chr1	223566715	223568812	C1orf65	164127	coiled-coil domain containing 185 [Source:HGNC Symbol; Acc:HGNC:26654]	protein coding	7	2	0.62	0.6	0.022	0.05	2871	No
ENSG00000251387	chr5	139152113	139154991	CTB-35F21.3			lincRNA	1	1	0.055	0.047	0.0082	0.0015	3564	Yes
ENSG00000198976	chr1	1104385	1104467	MIR429	554210	microRNA 429 [Source:HGNC Symbol; Acc:HGNC:13784]	miRNA	2	2	0.84	0.87	-0.022	0.0042	4123	No
ENSG00000258412	chr14	96677197	96681155	RP11-404P21.5			sense intronic	1	1	0.82	0.84	-0.018	0.038	5763	No
ENSG00000247993	chr5	72742184	72742811	FOXD1-AS1		FOXD1 antisense RNA 1 [Source:HGNC Symbol; Acc:HGNC:50658]	lincRNA	1	1	0.024	0.019	0.0053	0.021	6309	No
ENSG00000185069	chr12	53161939	53171129	KRT76	51350	keratin 76, type II [Source:HGNC Symbol; Acc:HGNC:24430]	protein coding	4	1	0.95	0.96	-0.013	0.026	10235	No
ENSG00000203942	chr10	99349450	99350690	C10orf62	414157	chromosome 10 open reading frame 62 [Source:HGNC Symbol; Acc:HGNC:23294]	protein coding	1	1	0.95	0.96	-0.011	0.0027	11576	No
