# Supplementary material for: Epigenomic profiling of preterm infants reveals DNA methylation differences at sites associated with neural function
Source: Transl Psychiatry. 2016 Jan 19;6(1):e716–. doi: 10.1038/tp.2015.210 (PMC5068883; doi:10.1038/tp.2015.210)
Supplement: Supplementary Table 2 [file tp2015210x2.pdf]

Table 2: Differentially expressed promoters, as derived by RnBeads

Ensembl ID	Chr	Start	End	Symbol	Entrez ID	Description	Gene type	No. sites	No. sites signif	Mean (0)	Mean (1)	Diff	p.fdr	Rank	DM CGI
ENSG00000232415	chr7	73476115	73478114	CTB-51J22.1			antisense	1	1	0.19	0.27	-0.084	0.00034	16	No
ENSG00000267385	chr19	4539580	4541579	CTB-50L17.14		Uncharacterized protein {ECO:0000313|Ensembl:ENSP00000464793} [Source:UniProtKB/TrEMBL; Acc:K7EIL1]	protein coding	5	2	0.58	0.48	0.096	0.00076	30	No
ENSG00000171236	chr19	4539987	4541986	LRG1	116844	leucine-rich alpha-2-glycoprotein 1 [Source:HGNC Symbol; Acc:HGNC:29480]	protein coding	4	2	0.58	0.48	0.096	0.00076	30	No
ENSG00000250020	chr5	56813	58812	RP11-811I15.1			processed pseudogene	1	1	0.61	0.48	0.13	6.5e-07	35	No
ENSG00000250280	chr12	65673937	65675936	RP11-305O6.3			sense intronic	1	1	0.29	0.39	-0.095	0.0037	40	No
ENSG00000164241	chr5	126408685	126410684	C5orf63	401207 101060408	chromosome 5 open reading frame 63 [Source:HGNC Symbol; Acc:HGNC:40051]	protein coding	14	11	0.3	0.36	-0.063	0.0013	55	Yes
ENSG00000252774	chr15	64633238	64635237	SNORA48		Small nucleolar RNA SNORA48 [Source:RFAM; Acc:RF00554]	snoRNA	1	1	0.13	0.23	-0.11	0.0086	55	No
ENSG00000136514	chr3	187084620	187086619	RTP4	64108	receptor (chemosensory) transporter protein 4 [Source:HGNC Symbol; Acc:HGNC:23992]	protein coding	5	2	0.68	0.57	0.1	0.00034	59	No
ENSG00000253837	chr8	23192184	23194183	RP11-177H13.2	100507156		processed transcript	1	1	0.16	0.1	0.061	0.00023	60	No
ENSG00000135406	chr12	49685535	49687534	PRPH	5630	peripherin [Source:HGNC Symbol; Acc:HGNC:9461]	protein coding	1	1	0.21	0.27	-0.061	0.00034	63	No
ENSG00000234502	chr9	104138386	104140385	FYTTD1P1		forty-two-three domain containing 1 pseudogene 1 [Source:HGNC Symbol; Acc:HGNC:38035]	processed pseudogene	1	1	0.53	0.43	0.1	0.00067	64	No
ENSG00000186910	chr14	94918628	94920627	SERPINA11	256394	serpin peptidase inhibitor, clade A (alpha-1 antiproteinase, antitrypsin), member 11 [Source:HGNC Symbol; Acc:HGNC:19193]	protein coding	1	1	0.44	0.53	-0.098	0.0019	66	No
ENSG00000167173	chr15	75486484	75488483	C15orf39	56905	chromosome 15 open reading frame 39 [Source:HGNC Symbol; Acc:HGNC:24497]	protein coding	1	1	0.28	0.34	-0.062	0.0057	71	No
ENSG00000261117	chr2	12854041	12856040	RP11-333O1.1			lincRNA	1	1	0.37	0.3	0.065	0.0012	76	No
ENSG00000108759	chr17	39623182	39625181	KRT32	3882	keratin 32 [Source:HGNC Symbol; Acc:HGNC:6449]	protein coding	5	3	0.56	0.49	0.065	0.00067	81	No
ENSG00000223125	chr17	39622708	39624707	RNU2-32P		RNA, U2 small nuclear 32, pseudogene [Source:HGNC Symbol; Acc:HGNC:48525]	snRNA	5	3	0.56	0.49	0.065	0.00067	81	No
ENSG00000252642	chr3	124776783	124778782	RNA5SP137		RNA, 5S ribosomal pseudogene 137 [Source:HGNC Symbol; Acc:HGNC:43037]	rRNA	1	1	0.53	0.64	-0.11	0.00076	85	No
ENSG00000160868	chr7	99381389	99383388	CYP3A4	1576	cytochrome P450, family 3, subfamily A, polypeptide 4 [Source:HGNC Symbol; Acc:HGNC:2637]	protein coding	4	2	0.44	0.49	-0.054	0.014	87	No
ENSG00000204020	chr10	90519663	90521662	LIPN	643418	lipase, family member N [Source:HGNC Symbol; Acc:HGNC:23452]	protein coding	1	1	0.2	0.14	0.059	0.018	87	No
ENSG00000218416	chr2	241395632	241397631	PP14571		uncharacterized LOC100130449 [Source:EntrezGene; Acc:100130449]	processed transcript	5	3	0.13	0.19	-0.053	0.00076	97	No
ENSG00000078098	chr2	163101162	163103161	FAP	2191	fibroblast activation protein, alpha [Source:HGNC Symbol; Acc:HGNC:3590]	protein coding	1	1	0.49	0.41	0.081	0.018	98	No
ENSG00000122641	chr7	41742207	41744206	INHBA	3624	inhibin, beta A [Source:HGNC Symbol; Acc:HGNC:6066]	protein coding	5	3	0.54	0.63	-0.085	0.00055	99	No

ENSG00000135063	chr9	71937988	71939987	FAM189A2	9413	family with sequence similarity 189, member A2 [Source:HGNC Symbol; Acc:HGNC:24820]	protein coding	2	2	0.37	0.42	-0.051	0.00067	111	No
ENSG00000250509	chr5	179867059	179869058	CTC-573N18.1			lincRNA	1	1	0.34	0.26	0.076	0.034	115	No
ENSG00000267457	chr17	33400894	33402893	RP5-837J1.4			lincRNA	1	1	0.094	0.14	-0.05	0.0012	115	No
ENSG00000207817	chr12	13067263	13069262	MIR614	693199	microRNA 614 [Source:HGNC Symbol; Acc:HGNC:32870]	miRNA	4	2	0.14	0.21	-0.071	0.034	116	No
ENSG00000260604	chr6	3911714	3913713	RP1-140K8.5			lincRNA	1	1	0.57	0.48	0.087	0.00034	122	No
ENSG00000169903	chr3	149190261	149192260	TM4SF4	7104	transmembrane 4 L six family member 4 [Source:HGNC Symbol; Acc:HGNC:11856]	protein coding	3	1	0.26	0.32	-0.057	0.05	143	No
ENSG00000169245	chr4	76944151	76946150	CXCL10	3627	chemokine (C-X-C motif) ligand 10 [Source:HGNC Symbol; Acc:HGNC:10637]	protein coding	4	1	0.61	0.52	0.088	0.05	145	No
ENSG00000204193	chr9	113099628	113101627	TXNDC8	255220	thioredoxin domain containing 8 (spermatozoa) [Source:HGNC Symbol; Acc:HGNC:31454]	protein coding	1	1	0.43	0.5	-0.072	0.033	147	No
ENSG00000145649	chr5	54396976	54398975	GZMA	3001	granzyme A (granzyme 1, cytotoxic T-lymphocyte-associated serine esterase 3) [Source:HGNC Symbol; Acc:HGNC:4708]	protein coding	4	1	0.11	0.16	-0.045	0.034	152	No
ENSG00000200135	chr12	112585664	112587663	Y RNA		Y RNA [Source:RFAM; Acc:RF00019]	misc RNA	1	1	0.32	0.37	-0.052	0.0036	160	No
ENSG00000069122	chr6	46922181	46924180	GPR116	221395	G protein-coupled receptor 116 [Source:HGNC Symbol; Acc:HGNC:19030]	protein coding	4	2	0.31	0.37	-0.062	0.0026	162	No
ENSG00000222112	chr1	33801966	33803965	RN7SKP16		RNA, 7SK small nuclear pseudogene 16 [Source:HGNC Symbol; Acc:HGNC:45740]	misc RNA	1	1	0.41	0.48	-0.065	0.00055	163	No
ENSG00000183729	chr8	53849491	53851490	NPBWR1	2831	neuropeptides B/W receptor 1 [Source:HGNC Symbol; Acc:HGNC:4522]	protein coding	1	1	0.67	0.58	0.091	0.0084	166	No
ENSG00000184330	chr1	153387500	153389499	S100A7A	338324	S100 calcium binding protein A7A [Source:HGNC Symbol; Acc:HGNC:21657]	protein coding	4	2	0.6	0.53	0.072	0.0051	169	No
ENSG00000109272	chr4	74717406	74719405	PF4V1	5197	platelet factor 4 variant 1 [Source:HGNC Symbol; Acc:HGNC:8862]	protein coding	6	5	0.32	0.36	-0.047	0.011	170	Yes
ENSG00000179409	chr17	656740	658739	GEMIN4	50628	gem (nuclear organelle) associated protein 4 [Source:HGNC Symbol; Acc:HGNC:15717]	protein coding	1	1	0.49	0.57	-0.076	0.00076	171	No
ENSG00000228176	chr1	30181895	30183894	RP4-656G21.1			lincRNA	1	1	0.43	0.37	0.055	0.031	191	No
ENSG00000162231	chr11	62573275	62575274	NXF1	10482	nuclear RNA export factor 1 [Source:HGNC Symbol; Acc:HGNC:8071]	protein coding	3	2	0.33	0.29	0.04	0.012	201	No
ENSG00000166949	chr15	67354601	67356600	SMAD3	4088	SMAD family member 3 [Source:HGNC Symbol; Acc:HGNC:6769]	protein coding	3	1	0.38	0.43	-0.057	0.0094	208	No
ENSG00000188394	chr9	125795306	125797305	GPR21	2844	G protein-coupled receptor 21 [Source:HGNC Symbol; Acc:HGNC:4476]	protein coding	4	2	0.5	0.54	-0.04	0.0049	212	No
ENSG00000213090	chr2	202277532	202279531	AC007256.5			processed pseudogene	1	1	0.5	0.57	-0.07	0.017	218	No
ENSG00000253302	chr8	74330739	74332738	STAU2-AS1	100128126	STAU2 antisense RNA 1 [Source:HGNC Symbol; Acc:HGNC:44101]	antisense	4	3	0.64	0.58	0.057	0.031	219	No
ENSG00000258297	chr11	66432007	66434006	RP11-658F2.8			antisense	1	1	0.35	0.4	-0.049	0.049	220	No
ENSG00000228789	chr6	31019727	31021726	HCG22	285834	HLA complex group 22 [Source:HGNC Symbol; Acc:HGNC:27780]	lincRNA	4	2	0.67	0.74	-0.067	0.049	231	No
ENSG00000270708	chr1	220392133	220394132	RP11-568G11.5			processed pseudogene	1	1	0.53	0.6	-0.071	0.017	241	No
ENSG00000261269	chr5	71737715	71739714	RP11-389C8.2			sense overlapping	2	1	0.61	0.69	-0.081	0.0049	245	No
ENSG00000105737	chr19	42573151	42575150	GRIK5	2901	glutamate receptor, ionotropic, kainate 5 [Source:HGNC Symbol; Acc:HGNC:4583]	protein coding	2	1	0.18	0.22	-0.038	0.0096	245	No

ENSG00000116251	chr1	6268950	6270949	RPL22	6146	ribosomal protein L22 [Source:HGNC Symbol; Acc:HGNC:10315]	protein coding	4	4	0.36	0.32	0.04	0.0029	277	Yes
ENSG00000205832	chr16	4604991	4606990	C16orf96	342346	chromosome 16 open reading frame 96 [Source:HGNC Symbol; Acc:HGNC:40031]	protein coding	3	1	0.73	0.65	0.082	0.016	283	No
ENSG00000254087	chr8	56790872	56792871	LYN	4067	LYN proto-oncogene, Src family tyrosine kinase [Source:HGNC Symbol; Acc:HGNC:6735]	protein coding	12	5	0.24	0.2	0.036	0.016	290	No
ENSG00000231177	chr3	10324603	10326602	LINC00852	84657	long intergenic non-protein coding RNA 852 [Source:HGNC Symbol; Acc:HGNC:29904]	antisense	5	2	0.7	0.76	-0.056	0.0048	296	No
ENSG00000259195	chr15	86312721	86314720	RP11-158M2.6			processed pseudogene	9	6	0.54	0.5	0.047	0.049	302	No
ENSG00000266233	chr3	98342389	98344388	AC021660.1			miRNA	2	1	0.45	0.5	-0.055	0.029	309	No
ENSG00000267319	chr19	38183966	38185965	CTD-2528L19.3			processed pseudogene	2	1	0.65	0.7	-0.046	0.018	319	No
ENSG00000103485	chr16	29688829	29690828	QPRT	23475	quinolinate phosphoribosyltransferase [Source:HGNC Symbol; Acc:HGNC:9755]	protein coding	3	1	0.14	0.18	-0.033	0.00067	331	No
ENSG00000248896	chr8	10585324	10587323	CTD-2135J3.3			antisense	10	6	0.3	0.27	0.033	0.03	332	No
ENSG00000207611	chr2	241393918	241395917	MIR149	406941	microRNA 149 [Source:HGNC Symbol; Acc:HGNC:31536]	miRNA	9	5	0.26	0.29	-0.032	0.014	348	No
ENSG00000222999	chr18	20715977	20717976	AC105247.1			miRNA	6	3	0.21	0.18	0.032	0.049	358	No
ENSG00000237115	chr6	132149054	132151053	RP1-131F15.2			processed pseudogene	1	1	0.88	0.8	0.089	9.7e-10	375	No
ENSG00000259321	chr14	24607154	24609153	RP11-468E2.5			lincRNA	1	1	0.5	0.56	-0.055	0.021	408	No
ENSG00000254789	chr11	15643438	15645437	RP11-531H8.2			lincRNA	1	1	0.71	0.79	-0.076	0.00034	421	No
ENSG00000253522	chr5	159893775	159895774	MIR146A	406938	microRNA 146a [Source:HGNC Symbol; Acc:HGNC:31533]	lincRNA	4	2	0.59	0.54	0.052	0.018	434	No
ENSG00000213128	chr17	78514503	78516502	RPL32P31		ribosomal protein L32 pseudogene 31 [Source:HGNC Symbol; Acc:HGNC:37035]	processed pseudogene	1	1	0.7	0.63	0.067	0.014	437	No
ENSG00000270937	chr6	1026961	1028960	RP5-856G1.1			lincRNA	1	1	0.82	0.75	0.078	5.3e-06	445	No
ENSG00000261293	chr16	12188665	12190664	RP11-276H1.2			antisense	1	1	0.6	0.67	-0.062	0.014	471	No
ENSG00000248884	chr5	67729809	67731808	CTC-537E7.3			lincRNA	2	1	0.062	0.033	0.028	0.00031	484	No
ENSG00000260498	chr16	87812481	87814480	RP4-536B24.4			lincRNA	5	3	0.3	0.27	0.027	0.049	539	No
ENSG00000258376	chr14	73712188	73714187	RP4-647C14.2			antisense	6	5	0.6	0.55	0.049	0.00076	551	Yes
ENSG00000164122	chr4	177198223	177200222	ASB5	140458	ankyrin repeat and SOCS box containing 5 [Source:HGNC Symbol; Acc:HGNC:17180]	protein coding	1	1	0.68	0.75	-0.065	0.038	552	No
ENSG00000147896	chr9	27522812	27524811	IFNK	56832	interferon, kappa [Source:HGNC Symbol; Acc:HGNC:21714]	protein coding	1	1	0.71	0.77	-0.067	0.018	569	No
ENSG00000267069	chr18	12286807	12288806	RP11-64C12.8			lincRNA	5	4	0.15	0.12	0.026	0.027	584	Yes
ENSG00000188511	chr22	50050691	50052690	C22orf34		chromosome 22 open reading frame 34 [Source:HGNC Symbol; Acc:HGNC:28010]	lincRNA	5	2	0.51	0.56	-0.045	0.018	596	No
ENSG00000225903	chr1	40097588	40099587	RP1-144F13.3			antisense	2	2	0.13	0.11	0.026	0.014	607	Yes
ENSG00000137135	chr9	35675364	35677363	ARHGEF39	84904	Rho guanine nucleotide exchange factor (GEF) 39 [Source:HGNC Symbol; Acc:HGNC:25909]	protein coding	3	2	0.23	0.21	0.026	0.0084	612	Yes
ENSG00000229257	chr9	139950615	139952614	RP11-229P13.22			antisense	1	1	0.77	0.71	0.064	0.014	614	No
ENSG00000226455	chr6	90658335	90660334	RP3-512E2.2			antisense	1	1	0.67	0.61	0.054	0.012	643	No
ENSG00000207935	chr9	73424501	73426500	MIR204	406987	microRNA 204 [Source:HGNC Symbol; Acc:HGNC:31582]	miRNA	2	1	0.69	0.75	-0.059	0.019	671	No
ENSG00000259725	chr16	54972084	54974083	CTD-3032H12.1			lincRNA	11	10	0.67	0.61	0.052	0.016	692	No
ENSG00000199961	chr17	74555691	74557690	SNORD1B	677849	small nucleolar RNA, C/D box 1B [Source:HGNC Symbol; Acc:HGNC:32676]	snoRNA	5	2	0.28	0.25	0.024	0.034	703	No
ENSG00000262810	chr17	1991475	1993474	RP11-667K14.5			antisense	1	1	0.8	0.74	0.061	0.023	735	No
ENSG00000269275	chr19	55888994	55890993	CTD-2105E13.15			antisense	5	2	0.74	0.69	0.054	0.018	743	No
ENSG00000233953	chr2	60721321	60723320	AC009970.1			antisense	2	2	0.65	0.7	-0.049	0.00031	793	No
ENSG00000206077	chr5	766568	768567	ZDHHC11B	653082	zinc finger, DHHC-type containing 11B [Source:HGNC Symbol; Acc:HGNC:32962]	protein coding	5	1	0.68	0.63	0.048	0.03	841	No
ENSG00000233085	chr6	169688812	169690811	XXyac-YX65C7 A.3			lincRNA	3	1	0.35	0.33	0.022	0.041	845	No

ENSG00000073605	chr17	38075608	38077607	GSDMB	55876	gasdermin B [Source:HGNC Symbol; Acc:HGNC:23690]	protein coding	2	1	0.75	0.79	-0.042	0.044	868	No
ENSG00000228680	chr7	43288368	43290367	AC004692.4			antisense	5	4	0.64	0.6	0.041	0.0022	890	Yes
ENSG00000110436	chr11	35441111	35443110	SLC1A2	6506	solute carrier family 1 (glial high affinity glutamate transporter), member 2 [Source:HGNC Symbol; Acc:HGNC:10940]	protein coding	10	4	0.19	0.17	0.021	0.014	904	No
ENSG00000236874	chr20	47013139	47015138	RP1-66N13.1			lincRNA	4	2	0.79	0.85	-0.056	0.034	927	No
ENSG00000199609	chr3	14434648	14436647	RNA5SP124		RNA, 5S ribosomal pseudogene 124 [Source:HGNC Symbol; Acc:HGNC:43024]	rRNA	1	1	0.7	0.66	0.046	0.038	997	No
ENSG00000249184	chr4	152719152	152721151	RP11-424M21.1			lincRNA	1	1	0.79	0.84	-0.054	0.019	1045	No
ENSG00000122043	chr13	30508542	30510541	LINC00544	440131	long intergenic non-protein coding RNA 544 [Source:HGNC Symbol; Acc:HGNC:43679]	lincRNA	1	1	0.63	0.59	0.04	0.014	1074	No
ENSG00000203786	chr1	152729006	152731005	KPRP	448834	keratinocyte proline-rich protein [Source:HGNC Symbol; Acc:HGNC:31823]	protein coding	6	2	0.33	0.36	-0.026	0.027	1095	No
ENSG00000178685	chr8	145086441	145088440	PARP10	84875	poly (ADP-ribose) polymerase family, member 10 [Source:HGNC Symbol; Acc:HGNC:25895]	protein coding	3	1	0.31	0.29	0.02	0.049	1107	No
ENSG00000103257	chr16	87902595	87904594	SLC7A5	8140	solute carrier family 7 (amino acid transporter light chain, L system), member 5 [Source:HGNC Symbol; Acc:HGNC:11063]	protein coding	8	1	0.75	0.71	0.046	0.0053	1129	No
ENSG00000068079	chr17	41157242	41159241	IFI35	3430	interferon-induced protein 35 [Source:HGNC Symbol; Acc:HGNC:5399]	protein coding	7	2	0.46	0.45	0.018	0.012	1191	No
ENSG00000114853	chr3	42693676	42695675	ZBTB47	92999	zinc finger and BTB domain containing 47 [Source:HGNC Symbol; Acc:HGNC:26955]	protein coding	6	3	0.2	0.21	-0.018	0.033	1230	No
ENSG00000129226	chr17	7481285	7483284	CD68	968	CD68 molecule [Source:HGNC Symbol; Acc:HGNC:1693]	protein coding	5	1	0.046	0.064	-0.018	0.038	1254	No
ENSG00000140398	chr15	75637796	75639795	NEIL1	79661 693216	nei endonuclease VIII-like 1 (E. coli) [Source:HGNC Symbol; Acc:HGNC:18448]	protein coding	5	2	0.38	0.4	-0.017	0.049	1301	No
ENSG00000116819	chr1	36037471	36039470	TFAP2E	339488	transcription factor AP-2 epsilon (activating enhancer binding protein 2 epsilon) [Source:HGNC Symbol; Acc:HGNC:30774]	protein coding	9	4	0.32	0.3	0.016	0.018	1436	Yes
ENSG00000265987	chr17	73338986	73340985	RP11-16C1.3			antisense	1	1	0.81	0.86	-0.046	0.0084	1471	No
ENSG00000111305	chr12	13256120	13258119	GSG1	83445	germ cell associated 1 [Source:HGNC Symbol; Acc:HGNC:19716]	protein coding	7	1	0.74	0.77	-0.036	0.049	1562	No
ENSG00000095970	chr6	41130425	41132424	TREM2	54209	triggering receptor expressed on myeloid cells 2 [Source:HGNC Symbol; Acc:HGNC:17761]	protein coding	6	2	0.76	0.73	0.026	0.029	1593	No
ENSG00000253673	chr5	157600904	157602903	CTC-436K13.1			lincRNA	1	1	0.91	0.87	0.046	0.00023	1616	No
ENSG00000258675	chr14	81916002	81918001	RP11-299L17.3	100506700		lincRNA	1	1	0.81	0.85	-0.042	0.0059	1694	No
ENSG00000159527	chr1	153282695	153284694	PGLYRP3	114771	peptidoglycan recognition protein 3 [Source:HGNC Symbol; Acc:HGNC:30014]	protein coding	6	2	0.84	0.89	-0.042	0.0075	1695	No
ENSG00000230080	chr11	1824826	1826825	AC139143.2			processed pseudogene	2	1	0.77	0.73	0.038	0.044	1711	No
ENSG00000252714	chr15	40623350	40625349	RNA5SP392		RNA, 5S ribosomal pseudogene 392 [Source:HGNC Symbol; Acc:HGNC:43292]	rRNA	2	1	0.79	0.82	-0.034	0.04	1765	No
ENSG00000235700	chr1	157096654	157098653	CYCSP52	360155	cytochrome c, somatic pseudogene 52 [Source:HGNC Symbol; Acc:HGNC:24393]	processed pseudogene	5	2	0.81	0.85	-0.037	0.0023	1979	No
ENSG00000260272	chr16	2544533	2546532	RP11-20I23.1		Uncharacterized protein {ECO:0000313|Ensembl:ENSP00000455547} [Source:UniProtKB/TrEMBL; Acc:H3BQ06]	protein coding	3	2	0.8	0.77	0.032	0.055	2020	No

ENSG00000168237	chr3	52319605	52321604	GLYCTK	132158	glycerate kinase [Source:HGNC Symbol; Acc:HGNC:24247]	protein coding	2	1	0.042	0.054	-0.012	0.00067	2280	No
ENSG00000233542	chr1	1946470	1948469	RP11-547D24.1			antisense	1	1	0.6	0.57	0.025	0.055	2293	No
ENSG00000253024	chr11	67162283	67164282	RNU6-1238P		RNA, U6 small nuclear 1238, pseudogene [Source:HGNC Symbol; Acc:HGNC:48201]	snRNA	4	2	0.7	0.73	-0.028	0.037	2301	No
ENSG00000206612	chr12	49050066	49052065	SNORA2A	677793	small nucleolar RNA, H/ACA box 2A [Source:HGNC Symbol; Acc:HGNC:32584]	snoRNA	4	1	0.86	0.89	-0.033	0.046	2358	No
ENSG00000225208	chr10	103069275	103071274	RP11-107I14.2			lincRNA	5	1	0.27	0.29	-0.022	0.048	2498	No
ENSG00000231394	chr7	55659970	55661969	RP11-310H4.3			lincRNA	1	1	0.77	0.74	0.031	0.0089	2538	No
ENSG00000133328	chr11	63330356	63332355	HRASLS2	54979	HRAS-like suppressor 2 [Source:HGNC Symbol; Acc:HGNC:17824]	protein coding	4	2	0.88	0.91	-0.031	0.0036	2597	No
ENSG00000240288	chr3	10325938	10327937	GHRLOS	100126793	ghrelin opposite strand/antisense RNA [Source:HGNC Symbol; Acc:HGNC:33885]	antisense	6	3	0.82	0.85	-0.032	0.018	2652	No
ENSG00000207574	chr8	145018948	145020947	MIR661	724031	microRNA 661 [Source:HGNC Symbol; Acc:HGNC:32917]	miRNA	16	9	0.65	0.68	-0.026	0.032	2763	No
ENSG00000128285	chr22	41073254	41075253	MCHR1	2847	melanin-concentrating hormone receptor 1 [Source:HGNC Symbol; Acc:HGNC:4479]	protein coding	5	1	0.8	0.82	-0.028	0.041	2772	No
ENSG00000161653	chr17	42080414	42082413	NAGS	162417	N-acetylglutamate synthase [Source:HGNC Symbol; Acc:HGNC:17996]	protein coding	11	4	0.15	0.14	0.0099	0.029	3022	No
ENSG00000159958	chr22	42322323	42324322	TNFRSF13C	115650	tumor necrosis factor receptor superfamily, member 13C [Source:HGNC Symbol; Acc:HGNC:17755]	protein coding	5	2	0.55	0.53	0.016	0.0097	3650	No
ENSG00000262343	chr17	77823410	77825409	RP11-353N14.3			lincRNA	1	1	0.87	0.9	-0.028	0.018	3892	No
ENSG00000144824	chr3	111449844	111451843	PHLDB2	90102	pleckstrin homology-like domain, family B, member 2 [Source:HGNC Symbol; Acc:HGNC:29573]	protein coding	4	1	0.89	0.87	0.026	0.018	4044	No
ENSG00000251387	chr5	139154492	139156491	CTB-35F21.3			lincRNA	2	1	0.062	0.055	0.007	0.03	4588	Yes
ENSG00000271792	chr5	139153390	139155389	CTB-35F21.5			lincRNA	2	1	0.062	0.055	0.007	0.03	4588	Yes
ENSG00000263401	chr5	173155581	173157580	AC008674.1			miRNA	1	1	0.75	0.77	-0.022	0.054	4595	No
ENSG00000244355	chr6	31681633	31683632	LY6G6D	58530	lymphocyte antigen 6 complex, locus G6D [Source:HGNC Symbol; Acc:HGNC:13935]	protein coding	8	5	0.47	0.5	-0.03	0.0053	4801	No
ENSG00000259364	chr15	40746339	40748338	RP11-64K12.9			antisense	1	1	0.89	0.92	-0.025	0.049	5000	No
ENSG00000253616	chr8	22931502	22933501	RP11-875O11.3			antisense	1	1	0.03	0.023	0.0061	0.052	5240	No
ENSG00000263013	chr16	11033402	11035401	RP11-876N24.5			sense intronic	1	1	0.81	0.83	-0.021	0.052	5446	No
ENSG00000262222	chr16	11033076	11035075	RP11-876N24.4	100505564		antisense	1	1	0.81	0.83	-0.021	0.052	5446	No
ENSG00000249661	chr4	141566144	141568143	TNRC18P1		TNRC18P1 [Source:HGNC Symbol; Acc:HGNC:43881]	processed pseudogene	1	1	0.93	0.9	0.022	0.0018	5990	No
ENSG00000172900	chr11	71133970	71135969	FLJ42102	399923	uncharacterized LOC399923 [Source:EntrezGene; Acc:399923]	unitary pseudogene	3	1	0.91	0.9	0.017	0.049	9013	No
ENSG00000238185	chr13	31551261	31553260	RP11-252M21.6			antisense	1	1	0.94	0.93	0.017	0.018	9086	No
ENSG00000079393	chr10	76868480	76870479	DUSP13	51207	dual specificity phosphatase 13 [Source:HGNC Symbol; Acc:HGNC:19681]	protein coding	5	1	0.87	0.89	-0.016	0.032	9156	No
ENSG00000259881	chr16	88945946	88947945	RP11-830F9.5			antisense	3	2	0.47	0.49	-0.025	0.014	9216	No
ENSG00000204422	chr6	31681350	31683349	XXbac-BPG32J3.20		Uncharacterized protein {ECO:0000313|Ensembl:ENSP00000475702} [Source:UniProtKB/TrEMBL; Acc:U3KQA8]	protein coding	8	4	0.44	0.47	-0.022	0.033	9221	No
ENSG00000255552	chr6	31681343	31683342	LY6G6E		lymphocyte antigen 6 complex, locus G6E (pseudogene) [Source:HGNC Symbol; Acc:HGNC:13934]	protein coding	8	4	0.44	0.47	-0.022	0.033	9221	No
ENSG00000260077	chr2	10180291	10182290	RP11-254F7.2			lincRNA	1	1	0.94	0.92	0.016	0.0031	9771	No
ENSG00000272476	chr6	108277117	108279116	RP1-191J18.66			antisense	1	1	0.018	0.015	0.0027	0.042	10114	No
ENSG00000207499	chr6	106867081	106869080	Y RNA		Y RNA [Source:RFAM; Acc:RF00019]	misc RNA	1	1	0.91	0.92	-0.015	0.054	10388	No

ENSG00000161010	chr5	179288674	179290673	C5orf45	51149	chromosome 5 open reading frame 45 [Source:HGNC Symbol; Acc:HGNC:30817]	protein coding	2	1	0.95	0.93	0.015	0.034	10430	No
ENSG00000199866	chr2	173422481	173424480	Y RNA		Y RNA [Source:RFAM; Acc:RF00019]	misc RNA	1	1	0.95	0.96	-0.011	0.0059	14277	No
ENSG00000115009	chr2	228677058	228679057	CCL20	6364	chemokine (C-C motif) ligand 20 [Source:HGNC Symbol; Acc:HGNC:10619]	protein coding	3	1	0.97	0.96	0.0064	0.046	18179	No
