# Supplementary material for: Epigenomic profiling of preterm infants reveals DNA methylation differences at sites associated with neural function
Source: Transl Psychiatry. 2016 Jan 19;6(1):e716–. doi: 10.1038/tp.2015.210 (PMC5068883; doi:10.1038/tp.2015.210)
Supplement: Supplementary Table 3 [file tp2015210x3.pdf]

Variance in DNAm of preterm infants explained by the first 23 principal components.

Principal component	Total variance / %	Cumulative variance / %
**1**	31.8	31.8
**2**	20.1	51.9
**3**	4.5	56.4
**4**	3.8	60.2
**5**	3.3	63.5
**6**	2.9	66.4
**7**	2.6	69.0
**8**	2.3	71.3
**9**	2.2	73.5
**10**	2.2	75.7
**11**	2.0	77.7
**12**	1.9	79.6
**13**	1.8	81.4
**14**	1.7	83.1
**15**	1.6	84.7
**16**	1.6	86.3
**17**	1.6	87.9
**18**	1.5	89.4
**19**	1.3	90.7
**20**	1.3	92.0
**21**	1.2	93.2
**22**	1.1	94.3
**23**	1.0	95.3

Values rounded to 1dp.
